# Rescuing biologically relevant consensus regions across replicated samples

**DOI:** 10.1186/s12859-023-05340-x

**Published:** 2023-06-07

**Authors:** Vahid Jalili, Marzia A. Cremona, Fernando Palluzzi

**Affiliations:** 1 Broad Institute of MIT and Harvard, Cambridge, MA USA; 2 Department of Operations and Decision Systems, Université Laval, Quebec, Canada; 3 CHU de Québec – Université Laval Research Center, Quebec, Canada; 4 Department of Brain and Behavioral Sciences, Università di Pavia, Pavia, Italy

**Keywords:** Peak calling, Replicated samples, Biological replicates, Technical replicates, Consensus regions, Weak binding affinities

## Abstract

**Background:**

Protein-DNA binding sites of ChIP-seq experiments are identified where the binding affinity is significant based on a given threshold. The choice of the threshold is a trade-off between conservative region identification and discarding weak, but true binding sites.

**Results:**

We rescue weak binding sites using MSPC, which efficiently exploits replicates to lower the threshold required to identify a site while keeping a low false-positive rate, and we compare it to IDR, a widely used post-processing method for identifying highly reproducible peaks across replicates. We observe several master transcription regulators (e.g., SP1 and GATA3) and HDAC2-GATA1 regulatory networks on rescued regions in K562 cell line.

**Conclusions:**

We argue the biological relevance of weak binding sites and the information they add when *rescued* by MSPC. An implementation of the proposed extended MSPC methodology and the scripts to reproduce the performed analysis are freely available at https://genometric.github.io/MSPC/; MSPC is distributed as a command-line application and an R package available from Bioconductor (https://doi.org/doi:10.18129/B9.bioc.rmspc).

**Supplementary Information:**

The online version contains supplementary material available at 10.1186/s12859-023-05340-x.

## Background

Chromatin immunoprecipitation (ChIP), followed by massively parallel DNA sequencing (ChIP-seq), has become the standard omics technique for the genome-wide localization of in vivo DNA–protein binding loci commonly known as “peaks”. A peak is generally inferred when the ChIP-seq read distribution differs significantly from the background signal, and its corresponding *p*-value is more stringent than a given threshold.

In general, peak evaluation is intrinsically limited due to the lack of annotations for “true” binding sites, especially for experimental conditions in which biological signals might be shifted, changed, or depleted [1]. Peak evaluation is complex, as gene expression regulation involves interactions between combinatorial transcription factor binding sites and chromatin states. Additionally, given the intrinsic noise of the ChIP-seq protocol that leads to artifactual peaks and poor localization of binding loci, peak calling methods are susceptible to high false-positive rates [2–4]. Peak callers utilize various methods to lower the false-positive rate. For instance, MACS (Model-based Analysis of ChIP-Seq, [2]) uses negative controls such as IgG (non-specific antibody-targeted ChIP-seq), and Ritornello [3] uses peak shape to differentiate between artifactual and “true” binding sites.

A common consensus suggests that “true” binding sites are reproducible across replicated samples (i.e., colocalized within a certain distance), whereas non-overlapping peaks are categorized as either of the following: (a) Characterizing “true” biological variability when studying biological replicates [5]; or (b) Artifactual binding or noise, particularly when studying technical replicates. Accordingly, replicated samples remain a reliable source of information to identify “true” binding sites [6]. However, calling such binding sites across replicated samples lacks gold standards, and it is associated with several open challenges, in particular for studying samples with low variability and high signal-to-noise ratio (technical replicates) and high variability with a low signal-to-noise ratio (biological replicates with heterogeneous cell populations [7]).

Multiple Sample Peak Calling (MSPC [8, 9]) and Irreproducible Discovery Rate (IDR [10]) are among the post-processing methods which can be applied to any peak caller results to identify reproducible peaks across replicated samples, representing “true” binding sites. IDR uses a copula mixture model to estimate the reproducibility of each pair of peaks in two replicates and to compute the expected rate of irreproducible discoveries [10]; it is considered the state-of-the-art for identifying reproducible peaks, being employed by the ENCODE consortium in their ChIP-seq processing pipeline. MSPC uses replicates to improve the sensitivity and specificity of peak calling on each sample. MSPC *rescues* weak peaks; in other words, it differentiates the weak binding sites which are reproducible across replicated samples from background signals (i.e., artifactual binding sites), taking into consideration if the replicates are biological or technical [8]. In this paper we perform an extensive evaluation of the biological relevance of the weak binding sites rescued by an extended version of MSPC, which greatly improves the computational efficiency of the original MSPC methodology, making it scalable and hence able to handle a very large number of replicates and a very large set of ChIP-seq experiments as the one considered in this study (a performance benchmark is given in Additional file 1: Results and Figs. S1–S2).

The binding sites that are highly reproducible across all the replicated samples are commonly referred to as *consensus regions*, and both MSPC and IDR can identify them. IDR ranks pairs of peaks in the two replicates based on their irreproducible discovery rate and combines those peaks with rates below a threshold. MSPC improves the sensitivity and specificity of each replicate and identifies their true-positive peaks using the Benjamini–Hochberg procedure; the extended version of MSPC then identifies consensus regions by merging the true-positive peaks and assigns each a combined stringency score (χ2 and right-tail probability).

The present study assesses the biological validity of the peaks MSPC (extended version) and IDR identify as “true binding sites” and the consensus regions they yield. Accordingly, we developed a novel feature enrichment test to prove the relevance of weak rescued peaks. Our results suggest that MSPC identifies more true binding sites and consensus regions than IDR, encompassing the IDR-identified regions in large. Additionally, our results show that the identified regions are enriched in biologically meaningful annotations and fully encompass essential information needed to understand genomic regulatory networks. For instance, using data from K562 cell line, we show the recovery of a large-scale enhancer regulatory network, depending on HDAC2 and GATA1 rescued peaks, whose components are involved in Chronic Myeloid Leukemia (CML) and several cancer-associated processes [11–16].

Identifying “true” binding sites (in noisy samples in particular) and consensus regions have a significant impact on studying high-throughput sequencing data [17, 18] with numerous applications spanning from improving sensitivity and specificity of peak callers to studying spatial dependency regulations and combinatorial transcription factor binding in different chromatin states [19–21]. The high-throughput sequencing data are available from public repositories such as ENCODE [22], Roadmap Epigenomics [23], and GEO [24], and are widely adopted for numerous biomedical studies. The quantity and quality of the reproducible regions identified in these samples can profoundly affect any downstream inferences. For instance, the high throughput sequencing data have been used for studying transcription factor regulatory networks [25–29], where identified peaks can vastly influence the topology and connectivity of the regulatory networks, including the inferred causal relationships [1]. Therefore, the results of the present study motivate utilizing methods such as MSPC and IDR to increase the specificity and sensitivity of peak callers and identify consensus regions.

## Material and methods

In the following, we first provide a brief literature review on the peak callers, we then discuss the characteristics of IDR and of the original version of MSPC, we describe the improvements implemented in the extended version of MSPC, and finally we define a novel functional enrichment test to assess the biological validity of the MSPC- and IDR-identified peaks.

### Characteristics of peak callers

A plethora of *peak calling* methods has been developed (reviewed in [1, 30–32]). In general, they differ in their statistical model and the number of input signals they operate on.

#### Statistical model

Peak callers identify binding affinities by either scanning the entire genome using a sliding window and test for differential binding between ChIP and control samples at each window based on the Poisson model or its extensions (e.g., MACS [2], PePr [33], and csaw [34]), or using a Hidden Markov Model approach (HMM, e.g., HPeak [35], ODIN [36], histoneHMM [37], and THOR [7]). The sliding window-based methods are sensitive to the window size, where large windows may fail to detect putative peaks (e.g., transcription factor binding sites) while narrow windows may generate severely fragmented peaks on wider binding sites (e.g., histone modifications). In general, methods using HMM can better detect subtle changes as they partition the signal into windows of varying sizes [7].

#### Number of input samples

Concerning the number of input samples, peak callers are generally divided into two groups. First group models binding affinity based on the signal of a single ChIP-seq assay (e.g., MACS). The second group jointly models binding affinities across replicated samples to identify combinatorial enrichment patterns (e.g. [19, 20, 33, 38–40]), they do so either by building models based on single samples then combine them (e.g., jMOSAiCS relies on MOSAiCS [38]), or based on HMM (e.g. [7, 37, 41]), or sliding window-based approaches (e.g., [33, 42–44]). In general, most differential peak calling methods are implemented using the sliding window approach, while a very few HMM-based approaches support replicated samples (e.g., THOR [7]). A possible shortcoming of the HMM-based approaches is that they model a ChIP-seq signal using a limited number of hidden states, which may result in less sensitivity to quantitative changes in signals of closely related conditions [45].

### Characteristics of MSPC and IDR

MSPC [8, 9] and IDR [10] are among the post-processing methods used to lower false-positive rates and identify consensus regions between replicated samples. Table 1 highlights the main characteristics of the extended version of MSPC (v6) presented in this paper and of IDR.

**Table 1:** Characteristics of the current extended version of MSPC (v6) and of IDR

	Model	Multiple Hypothesis Testing Correction	Replicate Count	Output Score
MSPC (v6)	Fisher’s combined probability test	False Discovery Rate (FDR, Benjamini–Hochberg procedure)	Unlimited	χ^2^, combined *p*-value
IDR	Gaussian copula mixture model	Local irreproducible discovery rate (idr)	2	Expected irreproducible discovery rate

IDR measures consistency between two replicates in high-throughput experiments. This quantitative irreproducibility score can then be used to rank pairs of peaks in the two replicates, determine a cutoff for irreproducibility and combine the two replicates. IDR uses a copula mixture model for estimating the expected irreproducible discovery rate of each pair of peaks in two replicates, yielding the expected rate of irreproducible discoveries [10].

Calling consensus regions using IDR falls short in two areas. First, IDR is developed for conservative peak detection, where only highly reproducible peaks across samples are called. Hence, it fails to call peaks in samples with large variance such as biological replicates, where strong peaks on one replicate do not colocalize with peaks from other replicates with a low signal-to-noise ratio (SNR) [1, 7]. Low SNR may not only arise due to poor sample quality, rather it can be reflective of true variability between biological replicates, low quantities of starting biological material, or antibody deficiency [46, 47]. Second, it can only deal with two replicated samples at a time, although increasing the number of replicates in ChIP-seq experiments is advisable to improve the analysis of these data [6], and several replicates could be harvested from separate consistent experiments [48] large cohort studies. Third, similar to other methods in this category, it relies on the candidate regions called by the peak caller, hence it may fail to detect subtle changes [18].

MSPC (both in its original and extended version) *rescues* weak peaks, addressing the first aforementioned shortcoming of IDR. It processes biological and technical replicates differently (see [8]), hence it differentiates between true variability between biological replicates and artifactual binding sites. Therefore, it lowers the false-negative rate between samples with large variance (expected in biological replicates) while preserving a low false-positive rate. Similarly to IDR, MSPC relies on the peak caller’s candidate regions; indeed, it consists in a post-processing step which can be applied to the results of any peak caller returning a *p *value for each of the peaks called. To alleviate this dependence, it is suggested to run MSPC on peaks called with a permissive *p *value threshold (e.g., 1e−4 [8];). Such a setting would lead to calling a large number of false-positives and a very small number of false-negatives, hence minimizing the probability of missing a true, yet weak binding site. MSPC uses combined stringency of peaks colocalized across replicated samples to differentiate between artifactual and weak binding sites, hence decreasing the number of false-negatives with least false-positives.

For each peak on a sample, MSPC finds the peaks in the other samples overlapping with it. If the number of overlapping peaks is more than a user-defined threshold, it then combines their *p *values using Fisher’s combined probability test, yielding a combined stringency, *χ*^2^, and the corresponding combined *p *value. MSPC *confirms* the overlapping peaks if the combined *χ*^2^ is larger than a user-defined threshold, and *discards* if otherwise. A peak might be tested multiple times if it overlaps with multiple peaks on another sample. Therefore, a peak might be *confirmed* based on one test and *discarded* based on another. When samples are biological replicates, MSPC *confirms* a peak if it passes at least one test (heterogeneity may reflect true biological variability), and with technical replicates, MSPC *discards* a peak if it does not pass all the tests (since more homogeneity is expected in this case). The confirmed peaks in each replicated sample are then corrected for false-discovery rate using the Benjamini–Hochberg procedure [8].

**Fig. 1:**
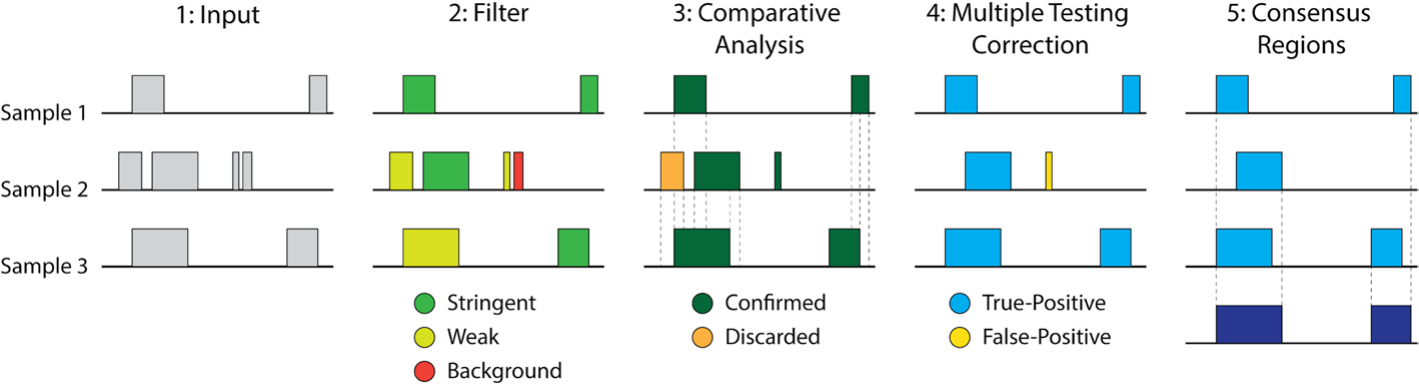
An illustration of processing regions on three replicated samples using the extended version of MSPC

One of the limitations of the original version of MSPC is that it returns a list of confirmed peaks for each replicated sample, leaving the user with the problem of combining replicates. The extended version of MSPC (v6) presented in this paper calls a consensus region where true-positive peaks on either of the replicates suggest binding loci. The coordinates of a consensus region are the union of overlapping true-positive peaks across all the samples, and its stringency is determined by combining the *p *values of the overlapping peaks using the Fisher’s combined probability test (see Fig. 1). Finally, the original version of MSPC is not computationally efficient, hence does not scale well with an increasing number of replicates. We greatly improved the computational aspects of the implementation of the extended version of MSPC. As a result, this extended version of MSPC enabled us to carry on the extensive analysis presented in this paper using reasonable computational resources; in addition, it can identify consensus regions across any number of replicates in a highly efficient and scalable way, hence addressing another limitation of IDR (see Table 1). A performance benchmark is given in the Additional file 1: Results and Figs. S1–S2.

Other post-processing methods for identifying reproducible peaks across replicates exist. For example, ChIP-R [48] represents an alternative to IDR which evaluates the reproducibility of peaks based on an adapted version of the rank-product test. A comparison among MSPC, IDR and ChIP-R is provided in the Additional file 1: Results and Figs. S3–S4.

### Data pre-processing

#### ENCODE data preprocessing

ChIP-seq raw data on K562 cell line were downloaded from ENCODE (see Additional file 1: Table S1); peaks on each sample were called using MACS2 with the following options: *–mfold 5, –bw 300 –pvalue 0.0001*. For each sample we used control samples as linked on ENCODE for each experiment; some experiments use a common control between multiple replicates (e.g., https://www.encodeproject.org/experiments/ENCSR532KTI/), some experiments use different control samples for each replicate (e.g., https://www.encodeproject.org/experiments/ENCSR121PFY/), or use two controls for each sample (e.g., https://www.encodeproject.org/experiments/ENCSR574XEO/).

#### Genomic annotations and optimal MSPC threshold set

Our functional enrichment procedure was applied to a set of 48 randomly chosen ENCODE transcription factors (TFs) for the cell line K562, as listed in the Additional file 1: Table S1. For each TF, the procedure was repeated using the extended version of MSPC (v6, see previous Section) and 10 different sets of MSPC thresholds (see Additional file 1: Table S2). The threshold sets were chosen from conservative to permissive, in order to cover different pools of rescued peaks. The best thresholds were defined as the ones producing the highest enrichment score (i.e., the highest z-score of the enrichment test, see details in the next Section) considering all TFs in the K562 analysis. The threshold set *−w 1E−04* (weak significance threshold), *−s 1E−08* (stringent significance threshold), and *−g 1E−06* (combined significance threshold), was the one yielding the best enrichment score for every TF. We then verified that this threshold choice leads to good results beyond the K562 cell line, by repeating the enrichment analysis for three TFs in MCF7 cell line (see Additional file 1: Results, Table S3, and Figs. S3–S4). To evaluate the enrichment for each TF, we selected 9 genomic annotations (genome assembly hg38), whose loci were downloaded from the UCSC Genome Browser database ([49] accessed on 2020-01-31): CpG islands, DNase clusters, enhancers, exons, introns, promoters, coding RefSeq genes, noncoding RefSeq genes, and non-RefSeq transcripts. We chose these annotations to have a straightforward measure of peak enrichment at open chromatin regions (DNase clusters), transcripts (exons, introns, coding RefSeq, noncoding RefSeq, and non-RefSeq transcripts), and both proximal and distal regulatory elements (promoters, CpG islands, enhancers).

### Functional enrichment test

The objective of the validation procedure is to assert if MSPC-rescued peaks are enriched in biologically meaningful loci. Accordingly, we defined three types of genomic regions: peaks, annotations, and regions not covered by any of them; where the first two may overlap to some extent. The higher the overlap between peaks and annotations, the higher the ability of the peak caller/rescuer to recall functional genomic regions. Since the number and coverage of a specific annotation is fixed for a given database, the only variables we need to consider are the number and position of called peaks. In particular, we define the conditional probability $p$ of a nucleotide overlapping a peak to contain an annotation, and the conditional probability $a$ of a nucleotide not overlapping a peak to contain an annotation (see Additional file 1: Methods for details). The difference $\beta =p-a$ measures the ability of the peak caller/rescuer to recall functional annotations, such that if $\beta$ > 0 (i.e., $p$ > $a$), there is a higher probability of observing a peak at a random position within an annotated region. Therefore, the greater the $\beta$ value, the higher the genome-wide proportion of annotated nucleotides within peaks. Our objective is to assert if the MSPC-rescued peaks on a given sample are strongly enriched in functional annotations w.r.t a standard baseline peak set given by IDR consensus on the same sample.

For both MSPC-rescued and IDR consensus peaks, we computed the enrichment score as the ratio $z =(\beta -{\beta }_{0})/\sigma$, where ${\beta }_{0}=0$ (i.e., $p=a$) is the $\beta$ value under the null hypothesis, and $\sigma$ is the standard error, that is the standard deviation of the sampling distribution of $\beta$, assuming that the underlying distribution of $z$ under the null hypothesis is well approximated by a gaussian distribution with mean 0 and standard deviation equal to 1 (see Additional file 1: Methods for further details). In this way, we can both assess the significance of annotation enrichment and directly compare the enrichment scores for MSPC-rescued peaks against IDR consensus, for each annotation and sample (i.e., transcription factor). Note that, although our enrichment test is based on a standard z-test approach, the definition of the conditional probabilities *p* and *a* and the resulting *z*-score are novel, in the sense that—to the best of our knowledge—they have never been used before for functional enrichment analysis. The scripts for the functional enrichment test are freely available from https://github.com/Genometric/MSPC/tree/dev/ValidationScripts.

### Overrepresentation analysis and motif search

TF binding motif enrichment was performed using MEME-ChIP with default settings [50] available at https://web.mit.edu/meme_v4.11.4/share/doc/meme-chip.html. Motif enrichment was evaluated using the threshold E-value < 1E−10.

Overrepresentation analysis against the KEGG pathways [51] and ChEA TF [52] databases was done using the Enrichr online tool [53] available at https://maayanlab.cloud/Enrichr.

### HDAC2-GATA1 enhancer regulatory network reconstruction

For each of the 48 TFs considered in this study, we obtained the list of transcription factor binding (TFB) motifs enriched at MSPC rescued enhancers (enrichment E-value < 1E−04; Additional file 2: Table S4). HDAC2 was the TF showing the strongest motif enrichment for another TF in the set of 48: GATA1. This means that HDAC2 rescued peaks at known enhancers are enriched in GATA1 binding motifs and, therefore, these enhancers are common HDAC2-GATA1 targets. Moreover, since the enrichment in GATA1 motifs is exactly at HDAC2 rescued peaks, common target enhancers should bind HDAC2-GATA1 simultaneously.

To further investigate the impact of this rescued regulatory network, we considered HDAC2 rescued enhancer peaks overlapping GATA1 rescued enhancer peaks, and considered the set of closest transcription start site (TSS) within 100 kb from these peaks, referred to as the HDAC2-GATA1 target gene set. We chose a 100 kb maximum distance to reduce enhancer-TSS false-positive associations, in accordance with recent literature [29, 54]. To evaluate the importance of the rescued HDAC2-GATA1 targets, we performed overrepresentation analysis (ORA) with three goals: (i) assess disease and pathway enrichment of the HDAC2-GATA1 target genes through the KEGG database (Additional file 3: Table S5 [51];); (ii) check if HDAC2-GATA1 target genes are enriched in transcriptional master regulators, and (iii) evaluate if these genes are regulatory targets in specific cell lines [52, 55]. We performed over-representation analysis (ORA) using the online enrichment analysis tool Enrichr [56].

In addition, we generated a graph of the portion of the rescued HDAC2-GATA1 regulatory network that can be confirmed through experimental evidence, co-expression analysis, and/or curated databases. Protein–protein connections were fetched from the STRING interaction database and retained only if the confidence score was at least 0.4. These filters allowed us to highlight strength and density of the known regulatory network underlying MSPC-rescued peaks.

## Results and discussion

### MSPC enrichment-based assessment

To have a reference set of reproducible peaks for each transcription factor, we run IDR 2.0.4 (available at https://github.com/nboley/idr) with a global IDR threshold of 0.05 from the output consensus regions. This assessment has two goals: (i) Verify the number of reference IDR peaks that are also detected by MSPC, and (ii) Assert if those MSPC-rescued peaks that are not in the IDR set are enriched in functionally important genomic regions (i.e., annotations). Notably, for every TF, the extended version of MSPC was able to find all the reproducible IDR peaks; we name them as the *common* set of peaks. Reproducible IDR peaks obtained with higher thresholds were also included in MSPC peaks (we performed an analysis experimenting with various IDR thresholds on a subset of three TFs from MCF7 cells; Additional file 4: Table S6). This result is explained by the fact that IDR aims to find a set of highly reliable peaks which are consistent among replicates, while MSPC aims to retain all strong peaks and rescue weak peaks based on the combined evidence coming from different replicates. The scripts to reproduce the performed analysis are available from https://github.com/Genometric/MSPC/tree/dev/ValidationScripts.

**Fig. 2:**
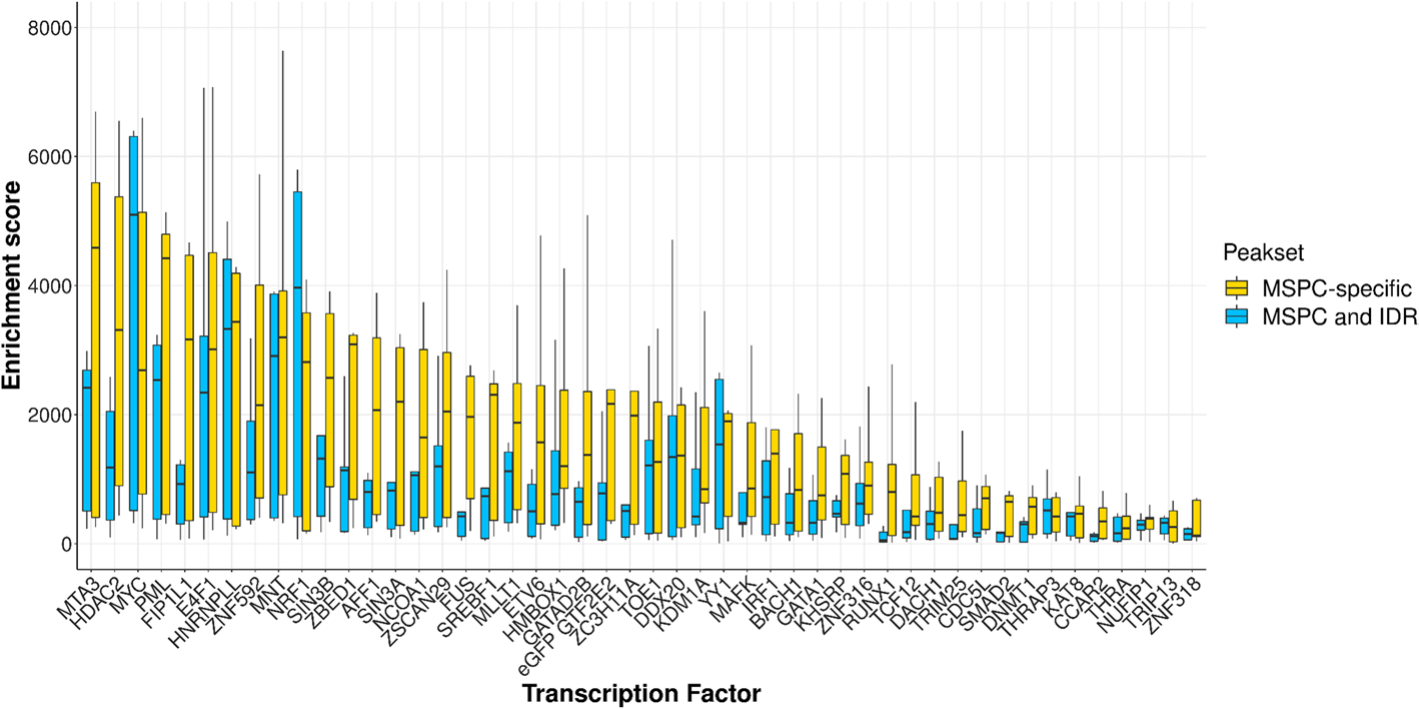
Enrichment score distribution (y-axis) for peaks retained by both MSPC and IDR (i.e., common peaks; cyan boxes) and rescued by MSPC but discarded by IDR (i.e., MSPC-specific peaks set; yellow boxes), aggregated by 48 ENCODE TFs in K562 cell line (x-axis). Note that there were no peaks retained by IDR and discarded by MSPC (i.e., MSPC always included IDR results). Among the 48 TFs analyzed, 44 showed higher median genomic annotation enrichments for MSPC-specific peaks (yellow boxes). The remaining 4/48 TFs (MYC, NRF1, THRAP3, and TRIP13) showed significant enrichment in genomic annotations (yellow boxes), although not higher than common peaks (cyan boxes). Note that a few outliers with enrichment scores greater than 8000 are included in the plot

**Fig. 3:**
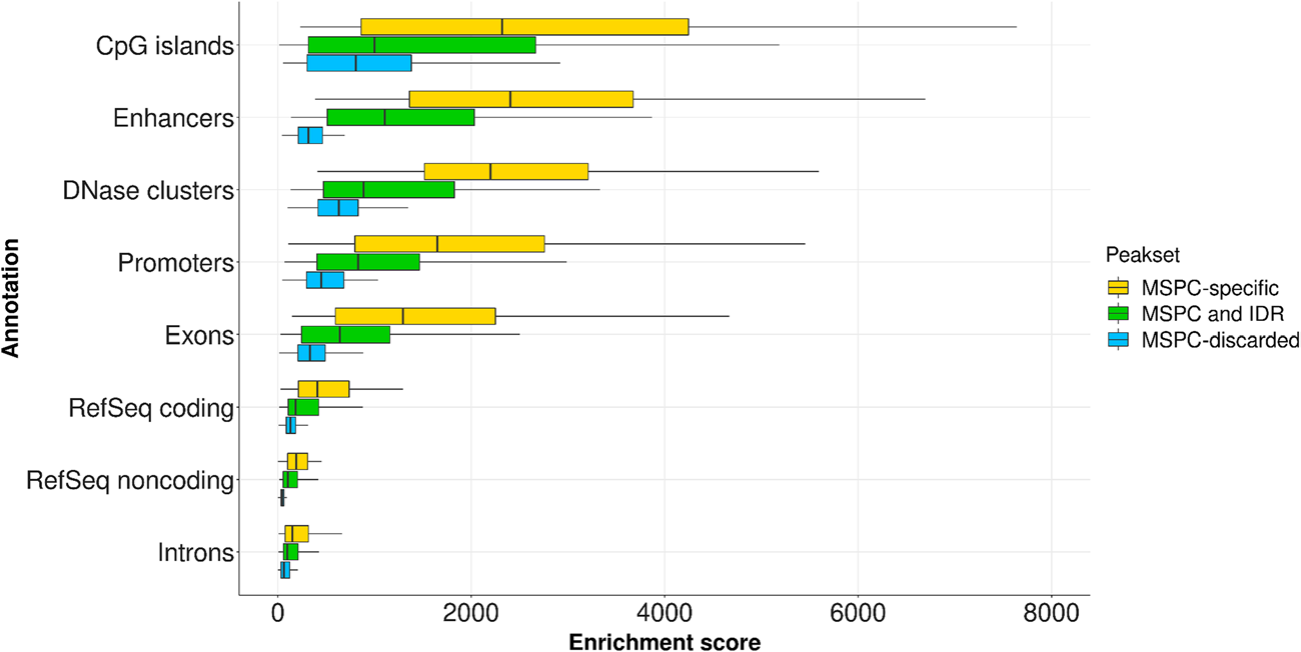
Enrichment score distribution (x axis) for MSPC discarded peaks (cyan box), MSPC rescued peaks discarded by IDR (i.e., MSPC-specific peaks set; yellow box), and peaks retained by both MSPC and IDR (i.e., common peaks; green box), aggregated by 9 hg38 annotations (y axis). For each of the 48 TFs analyzed in the K562 cell line, there were no peaks retained by IDR and discarded by MSPC (i.e., MSPC always included IDR results). The 9 hg38 annotations include: CpG islands (n = 31,144), Enhancers (n = 393,964), DNase clusters (n = 2,107,358), Promoters (n = 34,996), Exons (n = 313,276), RefSeq coding transcripts (n = 67,635), RefSeq non-coding transcripts (n = 17,271), Introns (n = 172,751). Coordinates for hg38 annotations were downloaded from the UCSC Genome Browser (accessed on: 2020-01-31) [49]

Rescued MSPC peaks not present in the common peak set are referred to as *MSPC-specific peaks set*. To assess the biological relevance of specific peaks, we compared their functional enrichment score (i.e., the z score of the enrichment test) to the score of the common set of peaks (Figs. 2 and 3). For every annotation, MSPC-specific peaks showed higher enrichment than common ones (Fig. 3). Accordingly, MSPC confirms reproducible peaks and rescues peaks whose functional role is not negligible. Notably, the most significant MSPC enrichments against common peaks were at enhancers, promoters, and DNase clusters, denoting MSPC’s best performances in rescuing critical regulatory and accessible chromatin regions. At the TF level, MSPC showed higher median enrichment in 44/48 TFs with respect to common peaks, meaning that it rescues critical genome-wide TF enrichments that would be lost otherwise. In addition, Additional file 5: Table S7 shows how MSPC generally retains much more peaks than IDR (median MSPC/IDR peak number ratio: 6.87), yet showing high enrichment for functional genomic elements (see the next sections for further examples and details), without altering peak length distribution (see Additional file 1: Fig. S5). This implies that the high stringency of IDR might hamper discovery, that is instead enabled by MSPC.

### Motif enrichment at rescued enhancers

MEME ChIP TFB motif enrichment at rescued enhancers shows the presence of several transcription master regulators. The most frequent enriched motif is GATA3 (20/48 TFs), which has been recently described as a key factor in enhancer-dependent cell reprogramming [12] and T-cell differentiation [13]. The second most frequent motif (19/48 TFs) is SP1, known for binding enhancers, regulating chromatin looping [57] and playing a key role in malignant hematopoiesis, through its interaction with GATA1 [11]. The critical role of chromatin looping is also demonstrated by the occurrence of CTCF binding site enrichments (the third-most enriched motif, with 18/48 TFs). CTCF is a widely studied insulator which is recognized as one of the main designers of topologically associated domains (TADs). TADs insulate portions of active chromatin, determining how and when genomic DNA is processed (e.g., transcribed and/or replicated). Although TADs are conserved among evolutionary-related species, cancer-associated cell fate reprogramming is often associated with mutated TAD boundaries [58]. Other cancer-associated TFB motif enrichments have been found as well, including RUNX1-RUNX3 involved in lymphoid cell differentiation [14, 15], and ETV6, involved in lymphoid malignant transformation [16]. The complete table of TFB motif enrichments at rescued enhancers is reported in Additional file 2: Table S4.

### HDAC2-GATA1 rescued regulatory network

For many transcription factors, the biological enrichment of the peaks rescued by MSPC is exceptionally higher than those considered as reproducible by IDR. This shows how peaks discarded by IDR could participate in biological processes that are actively involved in cell phenotype, and was confirmed by the over-representation analysis over Gene Ontology, KEGG, Reactrome, and ChEA. However, over-representation analysis cannot explicitly find which connections of the genome regulatory network are supported by MSPC-rescued elements. Therefore, we assessed the extent of the K562 regulatory network rescued by MSPC by directly reconstructing the interactions among annotated genomic elements.

GATA1-HDAC2 are among the most enriched transcription factors in MSPC-specific dataset, and are both known to be involved in leukemic transformation in chronic myeloid leukemia (i.e., the K562 cells phenotype). It is also known that TFs regulatory networks and their impact on gene expression modulation is highly dependent on the 3D structure of the DNA, which is in turn deeply influenced by enhancer activity. While a deep description of these mechanisms is out of the scope of this work, we anyhow assessed the presence of enhancer-related regulatory networks within the pool of MSPC-specific results, further highlighting the biological importance of MSPC-rescued peaks.

We identified 26,514 HDAC2 and 2,513 GATA1 enhancers (see Material and Methods, and Additional file 1: Methods) at MSPC-rescued binding loci (after removing peaks shorter than 200 bp); among them, 1627 peaks are in the *common* set. Over-representation analysis against the KEGG database (see Additional file 3: Table S5 [51];) confirmed Chronic Myeloid Leukemia (CML) as the most enriched pathway (adjusted *P *value = 1.37E−03), with 25 CML genes as targets of rescued enhancers. Other leukemia-related pathways were significantly enriched, including: VEGF signaling (19 target genes; adjusted *P *value = 5.19E−03), Calcium reabsorption (17 target genes; adjusted *P* value = 5.23E−03), Cell cycle (31 target genes; adjusted *P *value = 5.26E−03), Rap1 signaling (43 target genes; adjusted *P *value = 0.0186), Cellular senescence (35 target genes; adjusted *P* value = 0.0201), Platelet activation (28 target genes; adjusted *P *value = 0.0313), Leukocyte transendothelial migration (26 target genes; adjusted *P* value = 0.0314). In addition, GATA1 and GATA2 binding in K562 cells were the top overrepresented terms (adjusted *P *value: 9.04E−97 and 1.61E−86, respectively; Additional file 6: Table S8) among target genes, against the ChEA TF database [52], indicating how both rescued enhancers and their associated genes are targets of GATA1 regulatory network. Additional file 1: Fig. S6 shows the HDAC2-GATA1 portion of the network that is either experimentally proven or supported by curated databases, as well as the proteins known to be expressed in leukemic cells (which tend to occupy hubs and central nodes of the network). The resulting network consists of 464 nodes and 309 edges, showing an enriched connectivity (STRING PPI enrichment *P* value = 1.21e−09), indicating a non-random set of interacting nodes. Edges have a minimum STRING confidence score of 0.4 (the thicker the edge, the higher the score). In addition, leukemic genes showed significant enrichment in the regulatory network, with respect to the whole genome (58/949 known leukemia genes, STRING enrichment FDR = 5.63e−08).

To assess location and enrichment of GATA1 binding within these enhancers we ran Centrimo and Fimo (from the MEME-ChIP suite [59]) on a region of 1 kb surrounding each enhancer center, to show the whole motif enrichment probability profile. GATA1 resulted to be the top enriched motif within 100 bp (best Jaspar motif ID: MA0035.3, E-value = 1.5E−234, adjusted *P* value = 7.9E−238; Additional file 8: Table S10). Among 14,370 GATA1-enriched HDAC2 rescued enhancers, 3826 (26.6%) of them included this GATA1 motif (Additional file 9: Table S11). This percentage increases to 37% if we consider all motifs associated with GATA1 (Jaspar motifs ID: MA0035.3 and MA0140.2; MA0140.2 E-value = 2.5E−134; MA0140.2 adjusted *P* value = 1.3E−137). These percentages correspond roughly to those of GATA1 motif in HDAC2 peaks (9862 HDAC2 peaks with GATA1 motifs over 37,399 consensus peaks; E-value = 3.4E−898, adjusted *P* value = 1.6E−901). The percentage increases to 40% (i.e., 14,891/37,399) if we consider all enriched GATA1 motifs in HDAC2 peaks (Additional file 9: Table S11). To further validate the capability of MSPC to rescue GATA1 enriched peaks, we ran Centrimo and Fimo on GATA1 rescued peaks themselves, yielding 3827 GATA1-positive peaks over 9306 total consensus peaks (35.3%, E-value = 3.4E−898, adjusted *P* value = 1.6E−901; Additional file 9: Table S11). Also in this case, the percentage strongly increases (54%; i.e., 5014/9306) if we consider all GATA1 motifs (see Additional file 9: Table S11 for all frequencies and significance). For each of the above mentioned cases, GATA1 peaks are always centrally enriched within 100 bp from the peak center (Additional file 1: Figs. S7–S8 for GATA1 motifs in GATA1 peaks, and Additional file 1: Figs. S9–S10 for GATA1 motifs in HDAC2 peaks).

Collectively, these results show how MSPC may successfully recover genome-wide enrichments (i.e., peaks) that are part of the K562 CML regulatory networks, coherently with the sample cell line and phenotype.

## Conclusion

We argue the significant impact of improving the sensitivity and specificity while identifying binding affinities on high-throughput sequencing data by discussing the biological characteristics unveiled using weak but reproducible binding sites. Specifically, our main contribution is proving that these rescued peaks are enriched in biologically meaningful sites. This information emerges from the results provided by our novel functional enrichment test, which show overrepresentation of genomic elements, including promoters, CpG islands, enhancers, and DNase clusters (regions of open chromatin), suggesting that these weak but reproducible elements are part of large-scale active chromatin networks (e.g., active enhancers and transcribed genes). We showed how one of these largest rescued regulatory networks is represented by the enhancers enriched in HDAC2-GATA1 peaks, which neighboring genes are involved in chronic myeloid leukemia-associated processes and K562-specific regulation.

We discuss two methods for differentiating between weak and artifactual binding sites and calling consensus regions across replicated samples: MSPC and IDR. The MSPC method discussed here extends the first public release [8] in multiple facets. Specifically, it generalizes to assays beyond ChIP-seq while maintaining high precision and recall ration; it improves the computational performance and minimizes resource requirements, such that it scales efficiently to large cohorts of single-cell assays (e.g., Assay for Transposase-Accessible Chromatin using sequencing ATAC-seq). Additionally, the MSPC version presented here yields a single set of consensus regions for a set of input replicates, where the significance of each region is computed as the combined stringency of colocalized binding sites. An R package was also implemented and released on Bioconductor for this extended version of MSPC.

Our analysis over K562 and MCF7 ENCODE data showed that MSPC contains all IDR-identified reproducible regions, in addition to “rescuing” many other biologically relevant weak regions. Although MSPC generally rescues much more peaks than IDR, we demonstrated that these peaks are highly enriched for functional genomic elements and TF binding motifs, showing how IDR approach is often too stringent, severely hampering discovery.

Additionally, MSPC consensus regions that are not common to IDR show a larger enrichment by annotation (8/8 genomic annotations; Fig. 3) and by TF (45/48 TFs; Fig. 2). Accordingly, the consensus regions identified by MSPC provide a more appropriate set of informative genomic regions, favoring discovery over conservativeness, while controlling false positives. Since MSPC is applied at post-peak calling, it can be used to produce a single set of peaks from multiple replicates, as well as from multiple sets of peaks obtained by applying different peak calling methods [60].

Both MSPC and IDR operate on regions called using peak callers. Hence, their candidate sites are limited to the regions identified by the peak caller. To alleviate this limitation, a recommended practice for MSPC is to call peaks using a permissive *p*-value threshold to minimize the probability of missing weak binding sites at the cost of increasing false-positive rate; our assessment shows that MSPC can distinguish between true weak binding sites and artifactual regions in an input with a high false-positive rate. Additionally, given that the statistical model of both methods rely on regions binding affinity, they have limited application in sequencing protocols where there is not sufficient evidence to reason about the statistical significance of binding affinity (e.g., single-cell protocols such as ATAC-seq).

## Supplementary Information

**Additional file 1. **Supplementary Tables, Figures, Methods and Results.

**Additional file 2.** **Supplementary Table 4**. This table shows the occurrence of transcription factor binding motifswithin MSPC-rescued enhancers associated with the 48 TFs. For each TF, a motif is considered to be enriched within enhancers and added to the table if the E-value reported by MEME-ChIPis below 1E-04.

**Additional file 3**. **Supplementary Table 5**. KEGG pathways enrichment. KEGG overrepresentation analysis was done using the Enrichr suite, using the list of genes in the neighborhoodof HDAC2-GATA1 associated enhancers. For each KEGG pathway, the following information is shown: number of genes of the list belonging to the pathway, P-value, Benjamini-Hochberg adjusted P-value, enrichment odds ratio, Enrichr combined score, symbols of genes in the list that belong to the pathway.

**Additional file 4**. **Supplementary Table 6**. IDR peak sets corresponding to different thresholds. The table reports the number of MSPC peaks with the chosen threshold setagainst several IDR thresholds for the TFs HDAC2, NRF1, and DDX20 in MCF7 cells. IDR peaks are always contained in the MSPC peak set.

**Additional file 5**. **Supplementary Table 7.** Peak counts in each replicate, as well as for MSPC, IDR and ChIP-R results, for both K562 and MCF7 analyses.

**Additional file 6.** **Supplementary Table 8**. ChEA transcription factor enrichment. ChEA overrepresentation analysis was done using the Enrichr suiteusing the list of genes in the neighborhoodof HDAC2-GATA1 associated enhancers. For each TF in ChEA, the following information is shown: number of genes of the list belonging to the pathway, P-value, Benjamini-Hochberg adjusted P-value, enrichment odds ratio, Enrichr combined score, symbols of genes in the list that belong to the pathway.

**Additional file 7**. **Supplementary Table 9**. GATA1-HDAC2 regulatory network. Nodesare represented both as official gene names and taxon. Ensembl protein IDs. The table reports whether a node is known to be expressed in leukemia cellsor not. The combined score only considers the contribution of experimental evidence, coexpression analysis, and curated database informationto support the interactions, and it must be greater than or equal to 0.4. This ensures that only high-confidence interactions are included. Other scores are reported for completeness: homology relationship, literature textmining, closeness on chromosome, gene fusion partners, phylogenetic relationship. Note that the table is redundant.

**Additional file 8**. **Supplementary Table 10**. Centrimo analysis results on HDAC2 enhancers. The table reports motif ID, motif name, consensus sequence, E-value and adjusted P-value for each detected motif. The strongest motif is GATA1.

**Additional file 9**. **Supplementary Table 11**. Summary of Fimo results for GATA1 in K562 cells. The table contains three blocks of rows: GATA1 peaks, HDAC2 peaks, and HDAC2 enhancers. In all these cases the table reports the number of total peaks, the number of regions containing GATA1-associated motifs, Jaspar motifs ID, motif enrichment E-value and adjusted P-value.

## Data Availability

An implementation of the proposed extended methodology and the scripts to reproduce the performed analysis are freely available at https://genometric.github.io/MSPC/, MSPC is distributed as a command-line application, an R package available from Bioconductor (https://doi.org/doi:10.18129/B9.bioc.rmspc), and a C# library.

## Abbreviations

DNA: Deoxyribonucleic acid
ChIP-seq: Chromatin immunoprecipitation sequencing
MACS: Model-based Analysis of ChIP-Seq
MSPC: Multiple Sample Peak Calling
IDR: Irreproducible discovery rate
IgG: Immunoglobulin G
TF: Transcription factor
HMM: Hidden Markov Model
SNR: Signal-to-noise ratio
TFB: Transcription factor binding
TSS: Transcription start site
ORA: Over-representation analysis
TAD: Topologically associated domain
CML: Chronic Myeloid Leukemia
ATAC-seq: Assay for Transposase-Accessible Chromatin using sequencing
