# Supplementary material for: Rescuing biologically relevant consensus regions across replicated samples
Source: BMC Bioinformatics. 2023 Jun 7;24:240. doi: 10.1186/s12859-023-05340-x (PMC10246347; doi:10.1186/s12859-023-05340-x)
Supplement: Supplementary file 1 — Additional file 1. Supplementary Tables, Figures, Methods and Results. [file 12859_2023_5340_MOESM1_ESM.pdf]

Rescuing Biologically Relevant Consensus Regions Across Replicated Samples

Vahid Jalili[1], Marzia Angela Cremona[2,3], Fernando Palluzzi[4]

[1] Broad Institute of MIT and Harvard, MA, USA

[2] Department of Operations and Decision Systems, Université Laval, Canada

[3] CHU de Québec – Université Laval Research Center, Canada

[4] Department of Brain and Behavioral Sciences, Università di Pavia, Italy

Supplementary Material

Supplementary Tables

Supplementary Table 1. Summary of the ChIP-seq experiments used the main text of this paper. The experiments are randomly selected from the ones available from ENCODE on the immortalized K562 cell line.

Index	ENCODE Experiment ID	Target Gene	Replicate Count	Control Count
1	ENCSR000BLR	SIN3A	2	1
2	ENCSR000BQY	PML	2	1
3	ENCSR000EFR	ZC3H11A	2	1
4	ENCSR000EGD	BACH1	2	1
5	ENCSR000EGJ	MYC	2	1
6	ENCSR000EGL	IRF1	2	1
7	ENCSR000EGX	MAFK	2	1
8	ENCSR000EWF	YY1	2	1
9	ENCSR000EWM	GATA1	2	1
10	ENCSR000FCE	ETV6	2	2
11	ENCSR030TJP	DACH1	2	2
12	ENCSR051DXE	FUS	2	2
13	ENCSR075HTM	HDAC2	2	2
14	ENCSR086FZL	KAT8	2	1
15	ENCSR112RNT	HNRNPLL	2	2
16	ENCSR121PFY	CDC5L	2	2
17	ENCSR154EIH	TRIP13	2	2
18	ENCSR167KBO	ZNF316	2	2
19	ENCSR175SZH	ZSCAN29	2	2
20	ENCSR177DNR	FIP1L1	2	2
21	ENCSR189PYJ	SMAD2	2	2
22	ENCSR189TRZ	TCF12	2	2

Index	ENCODE Experiment ID	Target Gene	Replicate Count	Control Count
23	ENCSR213HBY	TOE1	2	2
24	ENCSR249BHQ	ZNF592	2	2
25	ENCSR264CZJ	THRA	2	2
26	ENCSR286PCG	ZBED1	2	2
27	ENCSR334HSW	ZNF318	2	2
28	ENCSR414TYY	RUNX1	2	2
29	ENCSR426URK	AFF1	2	2
30	ENCSR446LAV	DDX20	2	2
31	ENCSR512NLO	MNT	2	2
32	ENCSR532KTI	GTF2E2	2	1
33	ENCSR547LKC	GATAD2B	2	2
34	ENCSR574XEO	NUFIP1	2	2
35	ENCSR598GER	CCAR2	2	2
36	ENCSR657JLK	SIN3B	2	2
37	ENCSR664AOA	TRIM25	2	2
38	ENCSR675LRO	MLLT1	2	2
39	ENCSR686EYO	KHSRP	2	2
40	ENCSR731LHZ	E4F1	2	2
41	ENCSR757IIU	HMBOX1	2	2
42	ENCSR815ZDS	SREBF1	2	2
43	ENCSR871TKJ	THRAP3	2	2
44	ENCSR908CMW	KDM1A	2	2
45	ENCSR914NEI	MTA3	2	2
46	ENCSR931HNY	NCOA1	2	2
47	ENCSR987PBI	DNMT1	2	2
48	ENCSR998AJK	NRF1	2	2

Supplementary Table 2. The sets of MSPC (v6) thresholds used in the experiments. The selected threshold (employed in the result section) is shown in bold.

	Weak Significance Threshold (-w)	Stringent Significance Threshold (-s)	Combined Stringency Threshold (-g)
1	1.00E-04	1.00E-05	1.00E-05
2	1.00E-04	1.00E-06	1.00E-05
3	1.00E-04	1.00E-06	1.00E-06
4	1.00E-04	1.00E-07	1.00E-07
**5**	**1.00E-04**	**1.00E-08**	**1.00E-06**
6	1.00E-04	1.00E-08	1.00E-08
7	1.00E-05	1.00E-06	1.00E-06
8	1.00E-05	1.00E-07	1.00E-07
9	1.00E-05	1.00E-08	1.00E-06
10	1.00E-05	1.00E-08	1.00E-08

Supplementary Table 3. Summary of the ChIP-seq experiments used in the section "MSPC enrichment-based assessment in MCF7 cell line" of the Supplementary results. The experiments are selected from the ones available from ENCODE on MCF7 cell line, as examples of highly, moderately, and poorly biologically-enriched MSPC-specific rescued peaks, respectively, in the main K562 analysis presented in the main text of the paper.

Index	ENCODE Experiment ID	Target Gene	Replicate Count	Control Count
1	ENCSR000BTP	HDAC2	2	4
2	ENCSR330ADN	DDX20	2	2
3	ENCSR135ANT	NRF1	2	4

Supplementary Table 4. This table shows the occurrence of transcription factor binding motifs (TFBMs) within MSPC-rescued enhancers associated with the 48 TFs. For each TF, a motif is considered to be enriched within enhancers and added to the table if the E-value reported by MEME-ChIP (either MEME or DREME algorithm) is below 1E-04.

Supplementary Table 5. KEGG pathways enrichment. KEGG overrepresentation analysis was done using the Enrichr suite (https://maayanlab.cloud/Enrichr/), using the list of genes in the neighborhood (< 100 kb) of HDAC2-GATA1 associated enhancers. For each KEGG pathway, the following information is shown: number of genes of the list belonging to the pathway, P-value, Benjamini-Hochberg adjusted P-value, enrichment odds ratio, Enrichr combined score, symbols of genes in the list that belong to the pathway.

Supplementary Table 6. IDR peak sets corresponding to different thresholds. The table reports the number of MSPC peaks with the chosen threshold set (weak: 1E-04, stringent: 1E-08, and combined: 1E-06) against several IDR thresholds for the TFs HDAC2, NRF1, and DDX20 in MCF7 cells. IDR peaks are always contained in the MSPC peak set.

Supplementary Table 7. Peak counts in each replicate, as well as for MSPC, IDR and ChIP-R results, for both K562 and MCF7 analyses.

Supplementary Table 8. ChEA transcription factor enrichment. ChEA overrepresentation analysis was done using the Enrichr suite (https://maayanlab.cloud/Enrichr/) using the list of genes in the neighborhood (< 100 kb) of HDAC2-GATA1 associated enhancers. For each TF in ChEA, the following information is shown: number of genes of the list belonging to the pathway, P-value, Benjamini-Hochberg adjusted P-value, enrichment odds ratio, Enrichr combined score, symbols of genes in the list that belong to the pathway.

Supplementary Table 9. GATA1-HDAC2 regulatory network. Nodes (proteins) are represented both as official gene names and taxon. Ensembl protein IDs (taxon 9606 = Homo sapiens). The table reports whether a node is known to be expressed in leukemia cells (leukemia = 1) or not (leukemia = 0). The combined score only considers the contribution of experimental evidence (experimental), coexpression analysis (coexpression), and curated database information (database) to support the interactions, and it must be greater than or equal to 0.4. This ensures that only high-confidence interactions are included. Other scores are reported for completeness: homology relationship (homology), literature textmining (textmining), closeness on chromosome

(chromosome), gene fusion partners (gene_fusion), phylogenetic relationship (phylogenesis). Note that the table is redundant (i.e., for each edge, there are two lines: A -> B and B -> A; this is conventional in defining a non-directed network).

Supplementary Table 10. Centrimo analysis results on HDAC2 enhancers. The table reports motif ID, motif name (alternative motif ID), consensus sequence, E-value and adjusted P-value for each detected motif. The strongest motif is GATA1(Jaspar ID: MA0035.3, E-value = 2.0E-234, adjusted P-value = 7.9E-238).

Supplementary Table 11. Summary of Fimo results for GATA1 in K562 cells. The table contains three blocks of rows (query field): GATA1 peaks, HDAC2 peaks, and HDAC2 enhancers. In all these cases the table reports the number of total peaks (total regions), the number of regions containing GATA1-associated motifs, Jaspar motifs ID, motif enrichment E-value and adjusted P-value.

Supplementary Figures

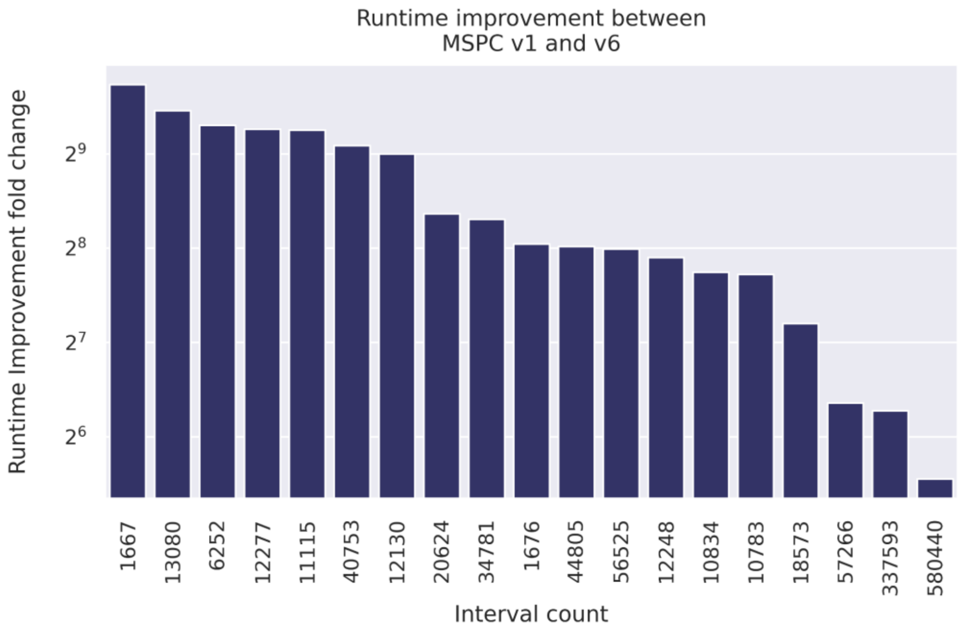

Supplementary Figure 1. Benchmarking results comparing MSPC v1 and v6, which show the fold change of the runtime (measured in wall-clock time and plotted in log2 scale) improvement between MSPC v1 (first version) and v6 (current latest release). This comparison is performed on a standard laptop with 4 cores, 16 GB RAM and solid-state drive storage, running Microsoft Windows 11 operating system.

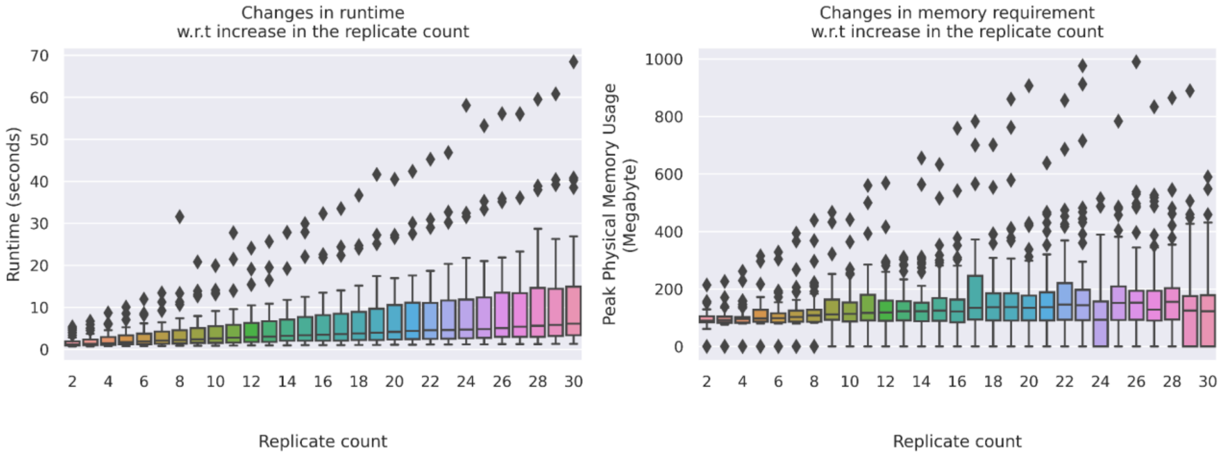

Supplementary Figure 2. Scalability of MSPC v6 with respect to the number of replicates. The left panel shows the scalability of runtime (measured in wall-clock time), and the right panel shows the scalability of memory requirements, measuring peak physical memory usage collected in intervals of every 100 milliseconds. This benchmark is performed on a standard desktop computer with 16 cores, 64 GB RAM, solid-state drive storage, and running Linux Ubuntu 22.04 operating system.

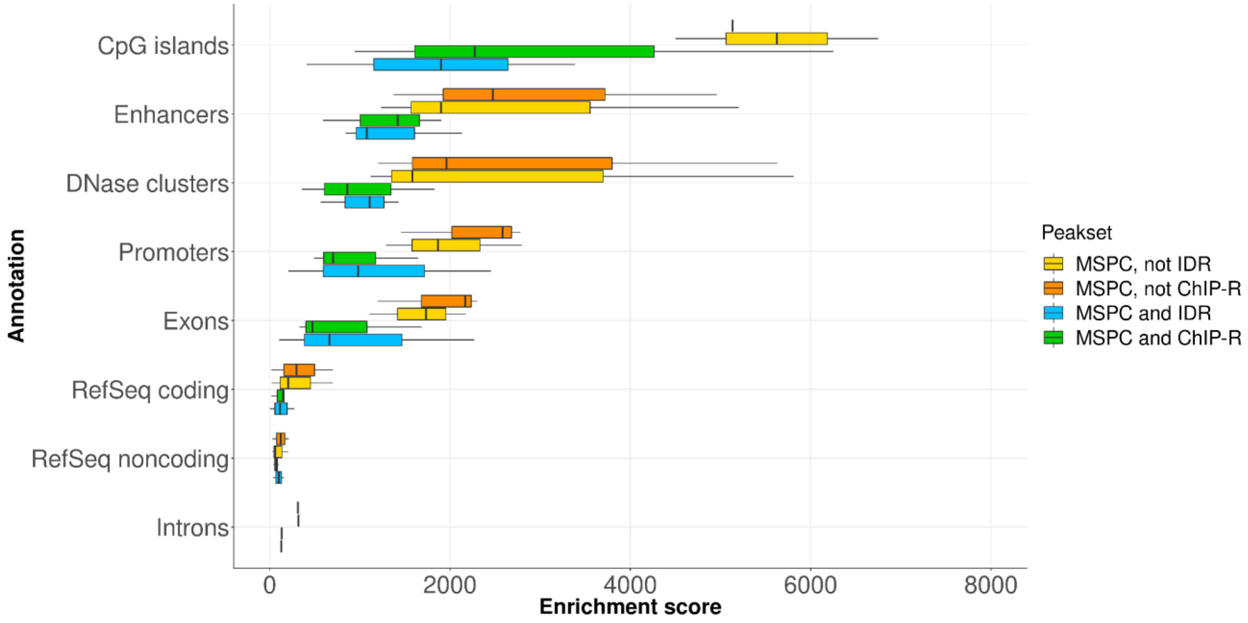

Supplementary Figure 3. Enrichment score distribution (y-axis) for peaks retained by both MSPC and IDR (cyan) or MSPC and ChIP-R (green), and peaks rescued by MSPC but discarded by either IDR (yellow) or ChIP-R (orange), considering three ENCODE TFs in MCF7 cell line.

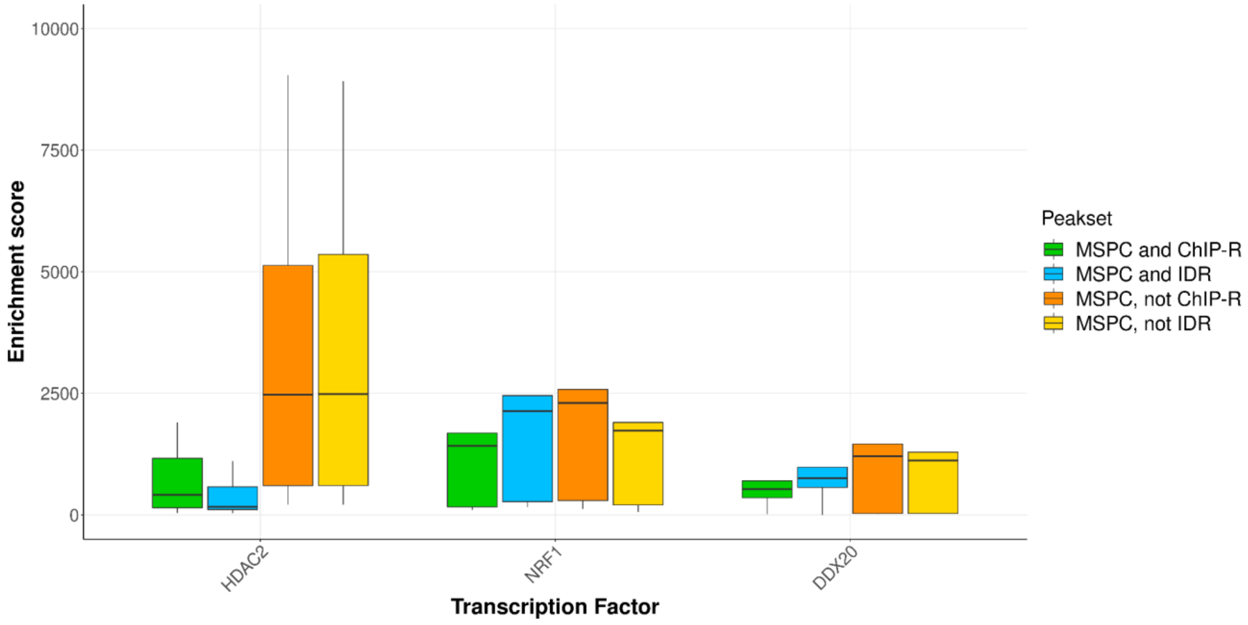

Supplementary Figure 4. Enrichment score distribution (y-axis) for common MSPC/IDR (cyan) and MSPC/ChIP-R (green) peaks, and MSPC peaks discarded by either IDR (yellow) or ChIP-R (orange), aggregated by three ENCODE TFs in MCF7 cell line (x-axis).

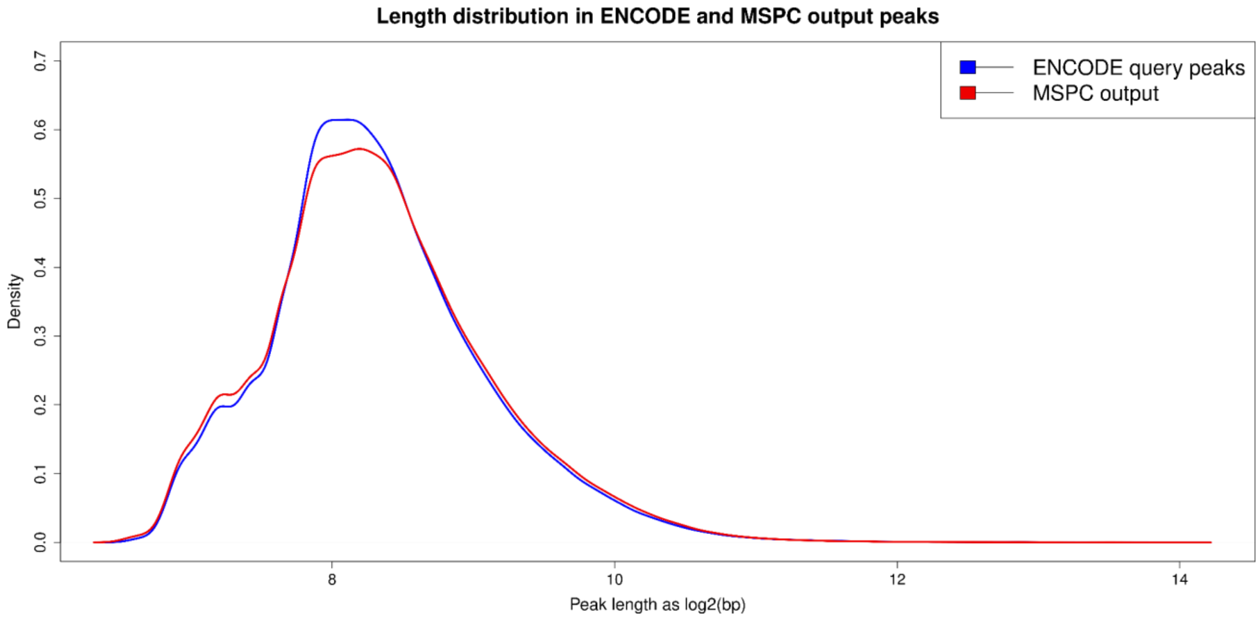

Supplementary Figure 5. Length distribution (in log2 scale, x axis) for ENCODE query peaks (blue distribution) and MSPC output peaks (red distribution). The figure clearly shows how MSPC processing does not affect peak length. ENCODE peak length quartiles: Q1 = 97 bp, median = 230 bp, Q3 = 432 bp. MSPC peak length quartiles: Q1 = 92 bp, median = 227 bp, Q3 = 441 bp.

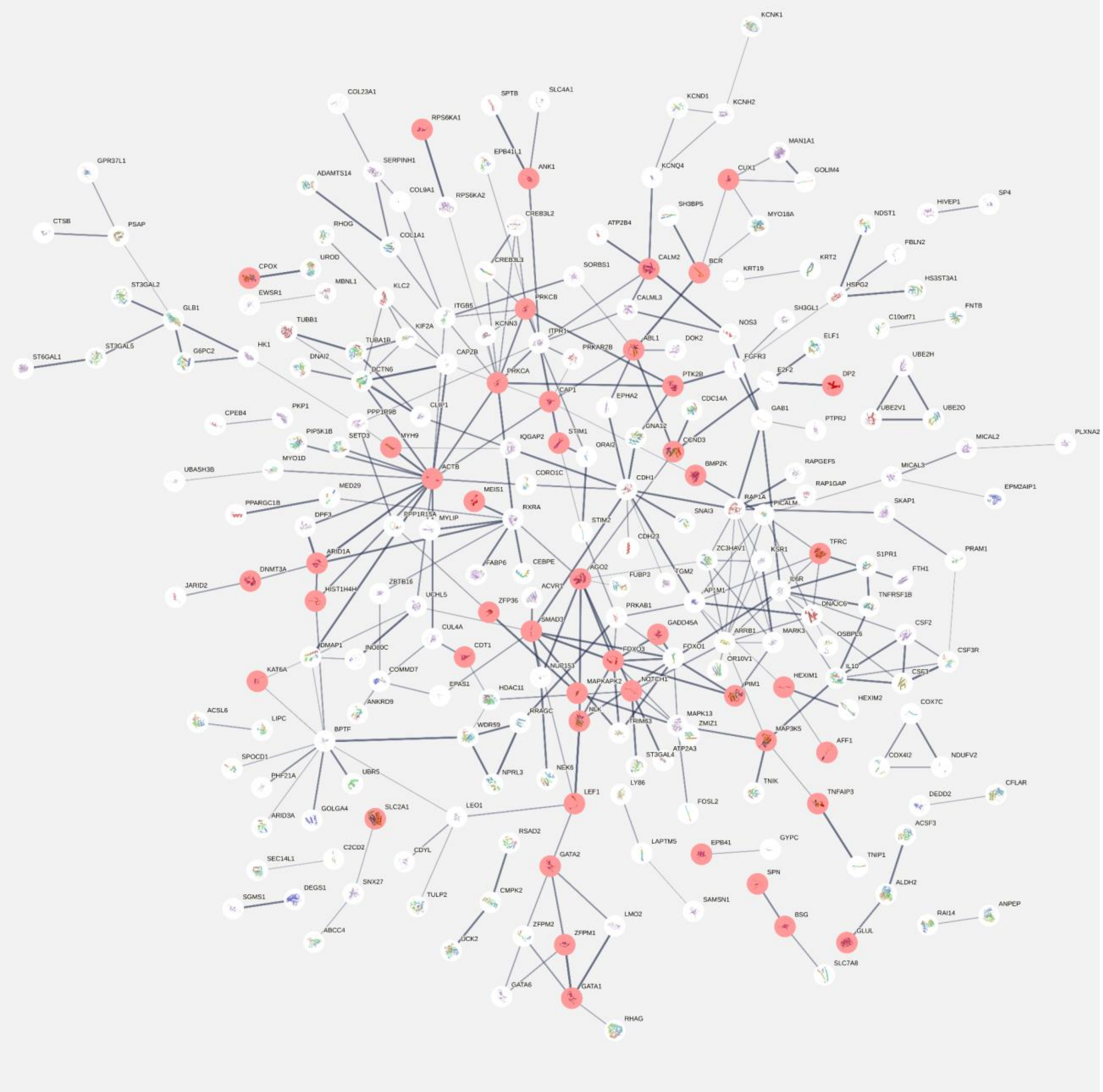

Supplementary Figure 6. HDAC2-GATA1 portion of the regulatory network. The network figure is generated using the STRING protein-protein interaction database, including connections from experimental evidence and/or curated databases, with a confidence score of at least 0.4. The thicker the edge, the higher the score. Red nodes show the proteins known to be expressed in leukemic cells.

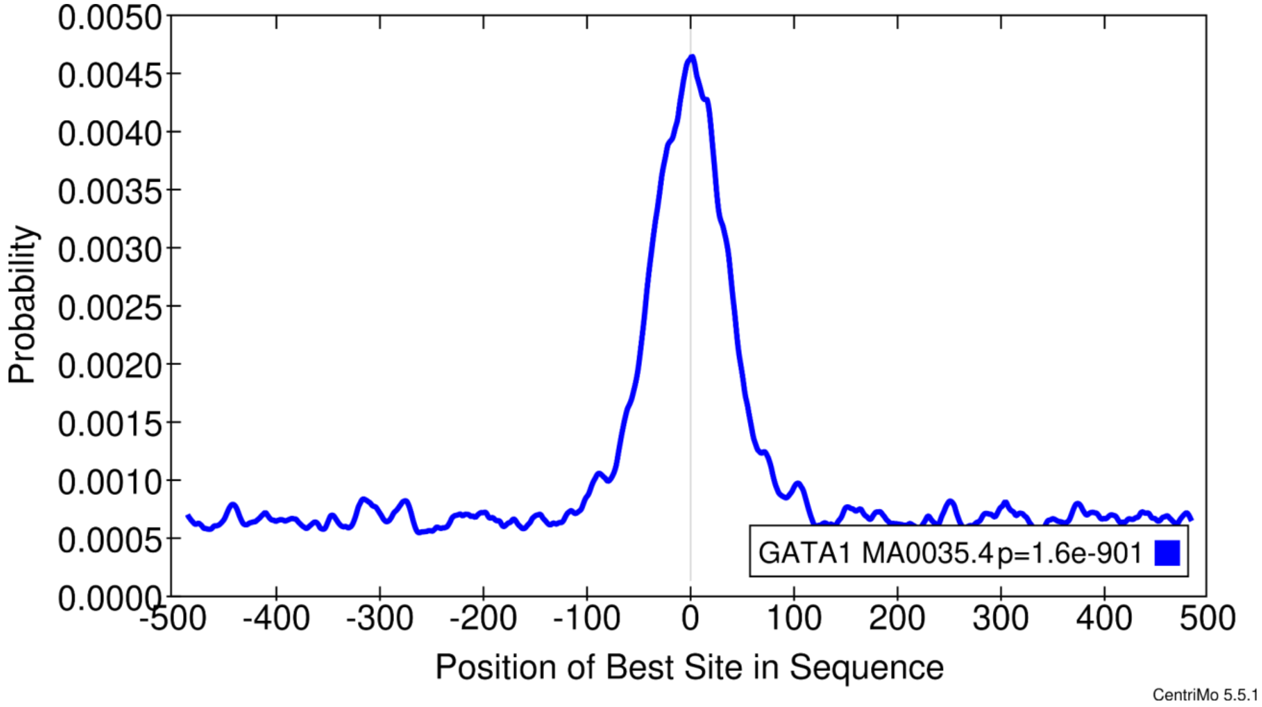

Supplementary Figure 7. Position of GATA1 Jaspar motif (MA0035.4) in GATA1 rescued peaks (Centrimo 5.5.1 adjusted P-value = 1.6E-901).

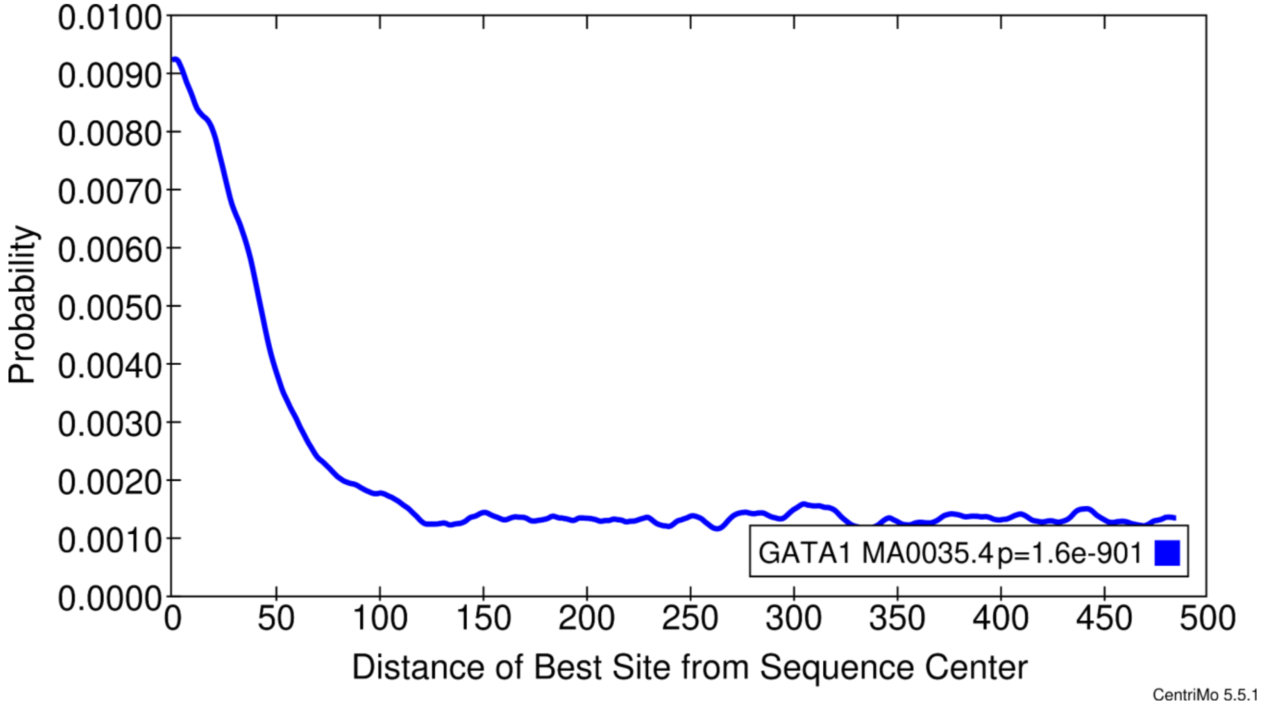

Supplementary Figure 8. Distance of GATA1 Jaspar motif (MA0035.4) in GATA1 rescued peaks (Centrimo 5.5.1 adjusted P-value = 1.6E-901) from peak center.

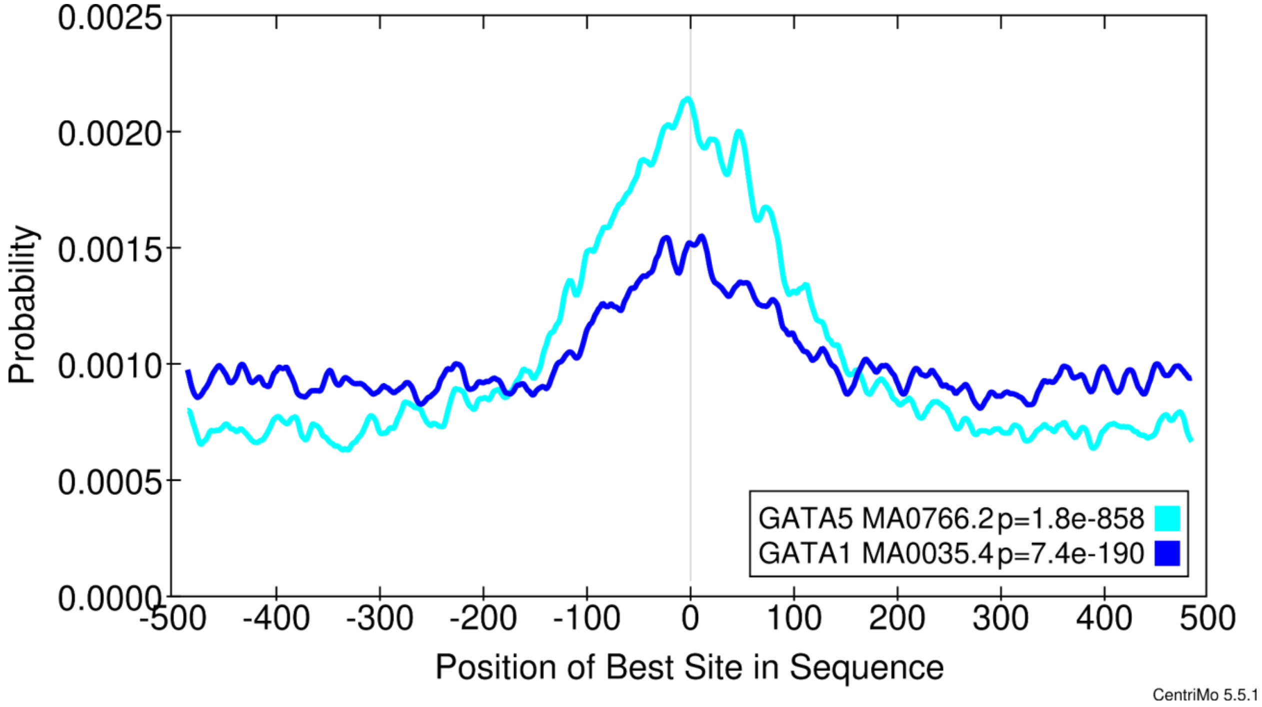

Supplementary Figure 9. Position of GATA1 Jaspar motif (MA0035.4) in HDAC2 rescued peaks (Centrimo 5.5.1 adjusted P-value = 7.4E-190, dark blue line). Enriched motif distribution is compared to the top enriched motif in HDAC2 peaks (GATA5, Jaspar motif ID: MA0766.2, adjusted P-value = 1.8E-858, cyan distribution).

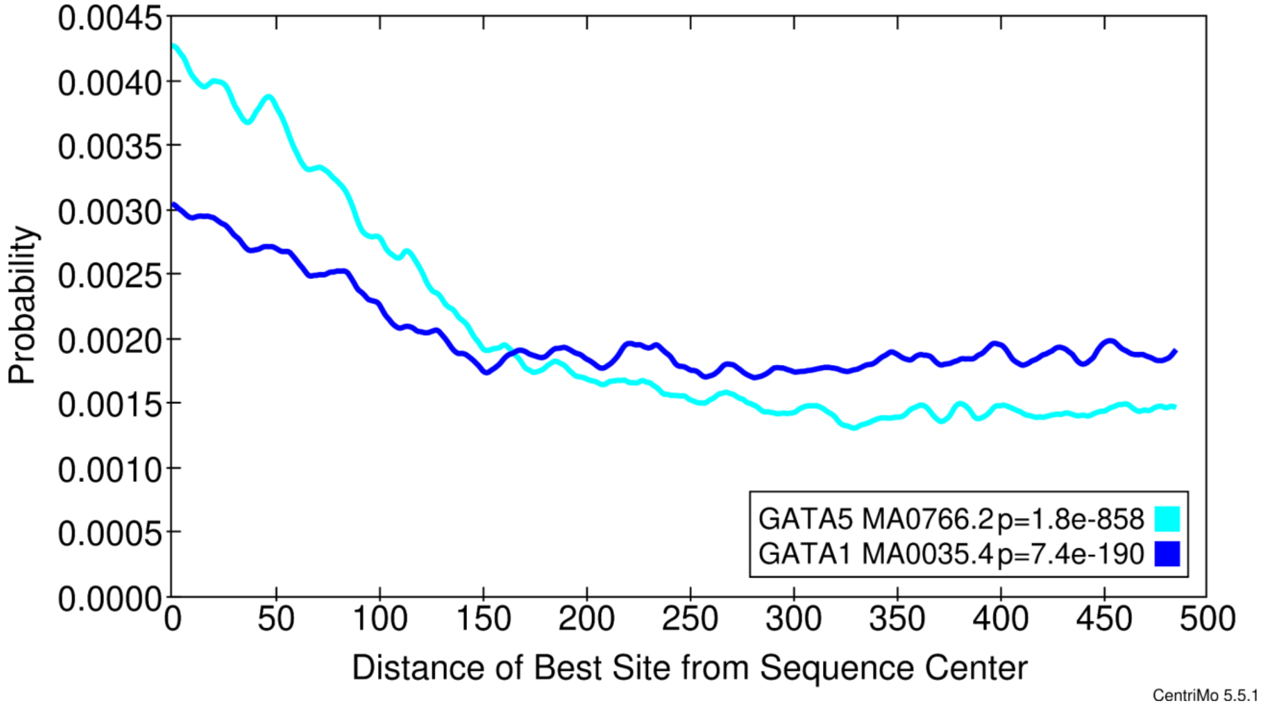

Supplementary Figure 10. Distance of GATA1 Jaspar motif (MA0035.4) in HDAC2 rescued peaks (Centrimo 5.5.1 adjusted P-value = 7.4E-190, dark blue line). Enriched motif distance is compared to the top enriched motif in HDAC2 peaks (GATA5, Jaspar motif ID: MA0766.2, adjusted P-value = 1.8E-858, cyan distribution).

Supplementary methods

Modeling of genomic annotations and peaks distribution

Let G denote genome; the null probability $P(A)$ of finding a functional annotation by randomly picking a single position in the genome can be estimated as A/G, where A is the size in bp (i.e. the genomic coverage) of the annotated portion of the genome. We define the binary random variable $Y \in \{0,1\}$ to indicate the annotations, i.e. we define $Y = 1$ if a nucleotide contains annotation and $Y = 0$ if the nucleotide lies outside of annotations. Similarly, we define the binary random variable $X \in \{0,1\}$ to indicate the nucleotides overlapping peaks found by the peak caller/rescuer. In particular, $X = 1$ if the nucleotide overlaps a peak, and $X = 0$ if it doesn't, and we indicate with U the total size of peaks in bp (i.e. the coverage of the union of all peaks).

We then consider the conditional probability of Y given X, i.e. the probability of a nucleotide to contain an annotation, conditionally of overlapping or non overlapping a peak. In particular, we define $p = P(Y = 1 \mid X = 1)$ the probability of a nucleotide overlapping a peak to contain an annotation, and $a = P(Y = 1 \mid X = 0)$ the probability of a nucleotide non-overlapping a peak to contain an annotation. We are interested in comparing p and a. We estimate the former with $\hat{p} = A_1/U$, where A_1 is the coverage of annotations within peaks (i.e. for which $X = 1$) and U is the coverage of the union of all peaks. Similarly, we estimate the latter with $\hat{a} = A_0/(G - U)$, where A_0 is the coverage of annotations outside peaks (i.e. for which $X = 0$) and $G - U$ is the amount of genome non-overlapping any peak.

Functional enrichment is expressed as the peak caller/rescuer capability of finding as many annotations as possible with the smallest possible number of peak calls. A large functional enrichment is achieved when $p = (Y = 1 \mid X = 1)$ is higher than $a = (Y = 1 \mid X = 0)$, therefore we model it as the proportion difference: $\beta = p - a$.

Functional Enrichment

Functional enrichment (β) is defined as the difference between the proportion of the genome converted by peaks that overlap annotations (p) and the proportion of the genome without peaks that overlap annotations (a):

$$\beta = p - a = P(Y = 1 \mid X = 1) - P(Y = 1 \mid X = 0)$$

Estimates:

$$\hat{\beta} = \hat{p} - \hat{a} \qquad \hat{p} = \frac{A_1}{U} \qquad \hat{a} = \frac{A_0}{G - U}$$

Where G is the genome size (bp count), U is the coverage of peaks (in bp), $A_0$is the coverage of annotations outside peaks (in bp), and $A_1$is the coverage of annotations within peaks (in bp).

Functional enrichment test

We test functional enrichment under the null hypothesis $H_0: \beta = 0$, versus the alternative $H_1: \beta \neq 0$. Note that, although we are generally interested in testing for positive enrichment (i.e. $\beta > 0$) for a given genomic feature, there are no strong assumptions allowing us to employ a one-sided test. For instance, under specific experimental conditions that may cause genome-wide repression of certain genomic elements, the peak caller/rescuer may find only a small number of active genomic regions (i.e., few peaks) and show feature under-representation (i.e.,$\beta < 0$). Significant feature under-representation could also arise within difficult genomic regions, such as repetitive or low complexity DNA, where it is hard to obtain reliable estimates of the signal-to-noise ratio and hence of peak presence.

Enrichment significance depends on the estimated effect size, i.e. on the difference $\beta - \beta_0$where β_0 is the null enrichment being tested (in our case, $\beta_0 = 0$), and on its standard error (σ). The standard error measures the reliability of the estimated effect size, depending on the proportions we are comparing and the sample size. Under the null hypothesis $H_0: \beta = 0$ we have $p = a$, so we can compute the pooled sample proportion as $\hat{q} = \left(\hat{p}U + \hat{a}(G - U)\right) / \left(U + (G - U)\right) = A/G$ and employ it to estimate σ as $\hat{\sigma} = \sqrt{\hat{q}(1 - \hat{q})\left(1/U + 1/(G - U)\right)}$. With constant U, as $\hat{q}$ approaches 0.5

the standard error is maximized. Conversely, if either $\hat{q}$ or $1-\hat{q}$ is a very small value, $\hat{q}(1-\hat{q})$ is small and so is the standard error. In our case, when the number of annotations is large (i.e. A is large) we obtain a small $\hat{\sigma}$. This happens because a peak corresponding to a regulatory (e.g., histone marker) or processing (e.g., RNA Polymerase II binding) signal, thus being neither a technical artifact nor random background fluctuation, is generally associated with a genomic region having a functional role (either active or repressed, depending on the signal type), that is more likely found within an annotation. Therefore, we expect a larger fraction of enriched peaks in a frequent genomic annotation (e.g., promoters), rather than a rare one (e.g., snoRNA), for which we expect higher a values.

The test statistic is $z = (\hat{\beta} - \beta_0)/\hat{\sigma}$, where β_0 is the effect size under H_0 (in our case, $\beta_0 = 0$). Under the null hypothesis, z is approximately distributed as a standard Normal $N(0,1)$, hence we can compute the p-value as the probability of observing a value as extreme as or more extreme than $|z|$in a standard Normal distribution, and we can compute the 95% confidence interval for β as $CI_{95\%} = (\hat{\beta} - 1.96\,\hat{\sigma};\ \hat{\beta} + 1.96\,\hat{\sigma})$. The $CI_{95\%}$ containing $\beta = 0$ (i.e., the null enrichment) is a synonym of non-significant enrichment at level 5%.

$$\hat{q} = \frac{\hat{p}U + \hat{a}(G-U)}{U + (G-U)} = \frac{A}{G}$$

Pooled sample proportion, with G: genome size and U: coverage of peaks

$$SE = \sqrt{\hat{q}(1-\hat{q})\left(\frac{1}{U} + \frac{1}{G-U}\right)}$$

Enrichment Standard Error)

$$Z = \frac{\hat{\beta} - \beta_0}{SE} \overset{H_0}{\sim} N(0,1)$$

Enrichment test statistic, in our case $\beta_0 = 0$ (i.e., no enrichment)

$$\text{p-value} = 2\left(1 - \phi(|Z|)\right)$$

Functional enrichment p-value ($\phi(\cdot)$ is the cumulative distribution of a standard Normal)

$$CI_{95\%} = \left(\hat{\beta} - 1.96\,SE, \hat{\beta} + 1.96\,SE\right)$$

95% confidence interval for β

TFB motif enrichment analysis at enhancers

To further evaluate the biological impact of MSPC rescued peaks, we assessed the presence of regulatory motifs at rescued enhancers. To ensure no overlap between MSPC and IDR results, we considered genomic features overlapping peaks rescued only by MSPC. We chose enhancers for two reasons. Firstly, enhancers have a clear and well-studied impact on gene expression, largely

documented on biological knowledge bases, including Gene Cards [60] and the UCSC Genome Browser [49], and providing a direct measure of the regulatory importance of rescued peaks. Secondly, part of an enhancer target gene set can be recovered by proximity to the closest TSSs [54], enabling a partial and yet robust evaluation of its impact on genomic regulation. To achieve this goal, we first isolated all MSPC rescued peaks overlapping at least one enhancer, from the hg38 GeneHancer set [60], downloaded from the UCSC Genome Browser (accessed on 2020-01-31). To improve the motif search, we excluded peaks shorter than 200 bp. The remaining peaks were used as input for transcription factor binding (TFB) motif search, using the MEME ChIP suite [61].

Supplementary results

Benchmarking MSPC improved performance and scalability

In addition to the functional improvements described in Material and Methods, we significantly improved MSPC performance and scalability. We benchmarked the current latest public release (v6, corresponding to the extended version described in this paper), against the version released when the method was first published (v1, [8]), using the replicates of the ENCODE ChIP-seq experiments listed in Supplementary Table 1. Since each of these experiments contains only two replicates, MSPC scalability was benchmarked using additional artificial replicates (i.e., replicates 3 to 30) generated by randomly altering the two available replicates.

The benchmarking results are given in Supplementary Figures 1-2, and show up to about 800x improvement in the runtime, as well as the scalability of the current MSPC version (both in runtime and memory usage) with respect to the number of replicates.

The scripts we employed for running the benchmarking are publicly distributed along with MSPC and available from https://github.com/Genometric/MSPC. Additionally, we have developed a Jupyter Notebook that runs in a Colaboratory environment, and publicly distributed it on MSPC's GitHub repository.

MSPC enrichment-based assessment in MCF7 cell line

We considered the ChIP-seq experiments for the transcription factors HDAC2, NRF1, and DDX20 available from ENCODE on MCF7 cells (see Supplementary Table 3). These three transcription factors were selected as examples of highly, moderately, and poorly biologically-enriched MSPC-specific rescued peaks, respectively, in the main K562 analysis presented in the paper (their ranking can be verified in Figure 2). HDAC2 is a master epigenetic regulator, for which we already presented an extended example of a rescued network in K562 cell (see Results). Regarding DDX20, the DEAD-box proteins have RNA-helicase activities that are not fully understood. Finally, NRF1 has a role as a respiratory metabolism regulator.

For each of these three TFs, we pre-processed the data following the same pipeline we employed for K562 experiments (see Material and Methods). Then, we run the extended version of MSPC

with the same thresholds we selected for the K562 dataset (-w 1E-04, -s 1E-08, -g 1E-06), and IDR 2.0.4 with a global IDR threshold of 0.05 (as in K562 experiments). In addition, for this analysis we also considered ChIP-R (Newell et al., 2021) with default options, as an alternative to IDR to assess the reproducibility of peaks. ChIP-R is an alternative to IDR, based on a rank-product test, which can evaluate the reproducibility from any number of replicates.

As shown in Supplementary Figure 3, there is a strong enrichment in transcriptionally-associated and regulatory elements of MSPC peaks, both against IDR and ChIP-R. Moreover, Supplementary Figure 4 reports how MSPC-rescued peaks are as enriched as or more enriched than the common peaks. Finally, Supplementary Table 7 includes all peak counts for both K562 and MCF7 analyses, showing how IDR and ChIP-R lead to similar enrichment levels, although their overlap (i.e., common peaks) ranges from 32 to 42%.
